# Epidemiological Assessment of African Swine Fever Spread in the Dominican Republic

**DOI:** 10.3390/pathogens12121414

**Published:** 2023-12-01

**Authors:** Rachel A. Schambow, Syed Hussain, Maria C. Antognoli, Silvia Kreindel, Raysa Reyes, Andres M. Perez

**Affiliations:** 1 Center for Animal Health and Food Safety, College of Veterinary Medicine, University of Minnesota, St. Paul, MN 55108, USA; hussa056@umn.edu (S.H.); aperez@umn.edu (A.M.P.); 2 International Services Action Programs, Animal Plant Health Inspection Service, United States Department of Agriculture, Fort Collins, CO 80526, USA; celia.antognoli@usda.gov (M.C.A.); silvia.kreindel@usda.gov (S.K.); 3 Instituto del Estudio de las Enfermedades Zoonóticas, Universidad Autónoma de Santo Domingo, Santo Domingo 10904, Dominican Republic; rreyes03@uasd.edu.do

**Keywords:** African Swine Fever, Dominican Republic, epidemiology, spatial, temporal, quantitative, biosecurity, pig, swine

## Abstract

Since African Swine Fever (ASF) was detected in the Dominican Republic in July 2021, it has negatively impacted the country’s swine industry. Assessing the epidemiological situation is crucial to helping local authorities and industry stakeholders control the disease. Here, data on 155 reported outbreaks in the Dominican Republic from November 2022 to June 2023 were evaluated. Descriptive spatiotemporal analysis was performed to characterize disease distribution and spread, and between-herd R_0_ was calculated for the study period. The Knox test and a space–time permutation model were used to evaluate clustering. Data on clinical presentation, biosecurity measures, and suspected reasons for introduction were categorized and summarized. The majority (78%) of outbreaks occurred on backyard farms which generally had low biosecurity. Across farm types, the majority of pigs were still alive at the time of depopulation. Spatiotemporal findings and R_0_ estimates suggest an endemic pattern of disease geographically located centrally within the country. Clustering was detected even at small temporal and spatial distances due to outbreaks amongst neighboring backyard farms. These results provide critical information on the current state of the ASF epidemic in the Dominican Republic and will aid government officials and swine industry leaders in developing effective ASF control strategies.

## 1. Introduction

Since its reintroduction into the Dominican Republic in 2021, African Swine Fever (ASF) has caused considerable disruption to the country’s swine industry. ASF is caused by the ASF virus (ASFV), a double-stranded DNA arbovirus of the family Asfiviridae that produces hemorrhagic fever in infected pigs [1,2]. ASFV only infects members of the Suidae family, including domestic pigs and wild boar, and does not affect humans nor is it a food safety threat. ASF is a reportable disease to the World Organization for Animal Health [3]. Since 1995, it was only known to be present in Africa and the island of Sardinia off the coast of Italy [3,4,5]. However, since 2007, ASF genotype 2 has been spreading throughout Europe, Asia, Southeast Asia, and in 2021, it has spread to the Dominican Republic and Haiti, which make up the island of Hispaniola [1,6,7,8]. As of the latest WOAH ASF Situation Report (published 14 August 2023), 50 countries have reported ASF occurrences since 2021, and 1.5 million domestic pigs have been lost to the disease or culling [9]. In many European countries, only or mainly wild boar have been affected by ASF [10]. Because outbreaks are not reported in many parts of the world, including Africa, the true number of ASF-caused deaths in pigs is likely underrepresented [9,10]. ASF is mainly managed by preventing introduction to farms and countries through high biosecurity, and infected farms are typically depopulated to prevent further disease spread [6].

ASF was first detected in the Dominican Republic in 1978 in the midst of a wave of global ASF spread [5,11]. In response, the Dominican Republic and Haitian officials, with military support, depopulated the island’s entire pig population to eradicate the disease. Hispaniola was free of ASF until it was detected again in July 2021 in the Dominican Republic and shortly after in Haiti in September 2021 [7,8]. Reports suggest that the initial introduction to the Dominican Republic may have occurred as early as April 2021 [12]. Currently, the Dominican Republic government employs a strategy of surveillance, depopulation, and indemnification of culled animals to support ASF control and eradication [13,14]. From 30 June 2021 to 30 September 2023, approximately $15.7 million USD was spent by Banco Agricola and $13 million by the United States Department of Agriculture Animal and Plant Health Inspection Service (USDA APHIS) on indemnification to affected producers in an effort to incentivize passive reporting of cases [15].

The swine industry is highly important to the Dominican Republic, being the second highest protein consumed following poultry and generating significant economic activity [13]. According to the 2022 census of the Ministry of Agriculture, the Dominican Republic has over 1300 moderate-to-high biosecurity commercial farms (Técnificadas and semi-téchnificadas), over 1600 small commercial farms (No técnificada), and approximately 13,000 backyard farms (transpatio) [16]. These backyard farms rely on pork production for economic support as living “piggy banks” and as a food source. Combined, the industry has an estimated inventory of over 500,000 head including all production stages as of December 2022 [16]. The Dominican Agribusiness Council estimated that the Dominican Republic swine industry has approximately $500 million USD in investments and generates around 58,000 jobs [13]. In 2022, local production was estimated to have decreased by 21% compared to 2021 because of the impact from ongoing ASF outbreaks [13].

Epidemiological analyses, including spatiotemporal techniques, can be highly useful in understanding ASF occurrences in the Dominican Republic and supporting the development of tailored prevention and control strategies. Spatial techniques, such as mapping and cluster detection, can help in identifying high-risk regions where ASF may be more prevalent, while temporal analysis allows for the identification of important trends in ASF prevalence [17,18]. Combined with descriptive analysis of the affected farms and their biosecurity measures, the potential reasons and risk factors for ASF introduction on farms can be better understood, ultimately aiding government officials and the swine industry in ASF control.

The objective of this work was to use descriptive epidemiology and spatiotemporal analysis to evaluate the ASF epidemic in the Dominican Republic, using data collected from November 2022 to June 2023. The study also aimed to identify potential biosecurity measures and reasons that may have contributed to ASF introduction on these farms. This work provides useful information on disease trends and producer behaviors for government officials and private sector stakeholders, ultimately to provide a better understanding of the current outbreak and to support ASF control efforts in the Dominican Republic. Additionally, these results will aid other ASF-affected and unaffected areas by providing important information for ASF preparedness and control.

## 2. Materials and Methods

### 2.1. Dataset

Data on farm outbreaks were made available from November 2022 to June 2023 as a part of ongoing government-operated outbreak verification, depopulation, and indemnity efforts. Outbreaks were identified through passive surveillance. According to WOAH, an outbreak is characterized as the identification of one or more cases within an epidemiological unit [19]. In the Dominican Republic, any premise containing pigs is defined as an epidemiological unit, i.e., any commercial or backyard farm, and an outbreak is defined as at least one ASF case identified on a farm.

Veterinary officers from the Dominican Republic Ministry of Agriculture collected the data analyzed here using a questionnaire (Supplementary File S1) as a part of the routine verification process of reported outbreaks. The data were not collected for the present study, and the questionnaire was designed by the veterinary authority for their use. At the time of their in-person visit to affected farms, veterinary officers collected information from affected producers and their site including date of the outbreak, location (latitude/longitude and administrative province, municipality, and section), farm type as defined below, clinical signs by production group (piglets, nursery, boar, sow, fattening), biosecurity measures related to farm and barn access, water source (recorded as aqueduct, river, stream, well, dam, or other), personnel and truck biosecurity, pig breeding and management, and an open-ended question asking the possible origin of the contagion according to producer. Farm types were consistent with those from the 2022 census [16], and were defined by the veterinary authority and are translated to English below (original versions are available in Supplementary File S1). Each site’s farm type classification was performed by the veterinary authority.

Familiar Traspatio or backyard farms: Farms without biosecurity and whose production is for family consumption and/or is marketed and consumed limited within the location. Maximum of 25 pigs including up to two sows.

Comercial No Técnificadas (commercial, non-technical, CNT): Commercial farms with little to no degree of technology and with little to no biosecurity, with more than 25 pigs or more than two sows.

Semi-técnificadas (semi-technical, ST): Commercial farms with a moderate degree of technology and a moderate level of biosecurity.

Técnificadas (technical): Commercial farms with a high degree of technology and a high level of biosecurity.

In total, data containing information on all 155 outbreaks reported and verified by veterinary officials were made available by the Dominican Republic Incident Command for ASF for analysis. Received data were cleaned and prepared for analysis by organizing into a generic database, removing duplicate observations, verifying latitude/longitude coordinates using ArcGIS Pro (version 3.1.3, ESRI Inc., Redlands, CA, USA) and province/municipality boundaries, and by translating Spanish comments into English with support from bilingual native Spanish speakers.

### 2.2. Descriptive Analysis

Descriptive analysis was performed in Microsoft Excel, version 2016, and R version 4.3.1. The mean, median, standard deviation, and range were calculated for pig inventory by farm type. An overall assessment of biosecurity level was not performed, but the presence/absence of individual measures as recorded by veterinary officials was summed for each farm type. Water sourced from aqueducts, wells, or other domestic water sources were categorized as “drinking water”, and water sourced from rivers and streams were categorized as “non-drinking water” (no farms were reported using dams). Open-ended responses (clinical signs and reason for introduction suspected by the producer) were analyzed using qualitative coding techniques [20]. Specifically, each text response was imported into Microsoft Excel and read, then an appropriate abbreviation of the comment, or a code, was written next to each response. A native Spanish speaker reviewed translations and codes. Codes were then reviewed and overlapping codes were combined into themes representing those ideas (e.g., “birds”, “cows”, “dogs”, and “horses” were combined into “non-pig animals”; or “movement of buyers” and “movement of workers” were combined into “People”). Codes were summed for the number of times they appeared by production type (clinical signs) and by farm (suspected reason). Clinical signs were reported by 103 farms (66%; missing information from 52 farms) and categorized into 14 types of signs. Suspected reasons were reported by 122 farms (79%; missing information from 33 farms) and condensed from an initial list of 22 codes down to eleven themes.

### 2.3. Descriptive Spatiotemporal Analysis

Two outbreaks (1% of all data) had missing date information and were omitted from temporal analyses (*n* = 153 with complete spatiotemporal information). An epidemic curve was produced by plotting the number of cases by week for the duration of the study period. For each week starting with week three, a three-week moving average was estimated by summing the current and preceding two weeks’ case count and dividing by three to produce a rolling average. Seasonal analysis was not performed due to having an insufficient time period of data (approximately eight months).

The locations of all outbreaks (*n* = 155) were plotted using ArcGIS Pro and summed by province to produce a choropleth map depicting the number of cases by province. The mean center of all outbreaks was also calculated. Map breaks were selected using the natural breaks (Jenks) setting with an additional category representing provinces where no outbreaks were reported. The exact locations of farms were not provided here to protect their confidentiality. Additional choropleth maps were produced using the same settings for approximately every three months of the period, as November–December 2022, January–March 2023, and April to June 2023. Two outbreaks (1 in Barahona and 1 in Santo Domingo provinces) did not have dates and thus could not be included in these approximately tri-monthly choropleth maps.

### 2.4. Cluster Detection

Global autocorrelation for all outbreaks was assessed in ArcGIS Pro using Moran’s I, a measure of overall clustering of spatial data [21,22]. The null hypothesis of the Moran’s I statistic states that the feature being analyzed is randomly distributed throughout the spatial study area, and values of Moran’s I statistic may fall between −1.0 (similar features are dispersed) and +1.0 (similar features are clustered). Moran’s I was estimated using the inverse distance setting (so that nearby neighboring features have a larger influence compared to those farther away) using a default threshold (the Euclidean distance, ensuring every feature has at least one neighbor) and Euclidean distance (distance estimated as a straight line between points).

ClusterSeer (v. 2.5, BioMedware, Ann Arbor, MI, USA) was used to evaluate the interactions between space and time and outbreak clustering using the Knox test (*n* = 153 due to 2 outbreaks with missing date information). The Knox test is a comparison of the proportion of cases in the study population that occurred in close proximity in both space and time to the proportion expected if cases were equally and homogenously distributed throughout the study space and time period [23]. Values of 1, 3, 5, 10, 15, 20, and 30 km and 1, 3, 5, 7, 9, 14, 21, and 28 days were used as critical values for spatial and temporal closeness, respectively. All combinations were analyzed using the Knox test to obtain their associated *p*-value (estimated via the generation of 999 Monte Carlo simulations) and observed-to-expected (O/E) ratio.

### 2.5. Space–Time Permutation Model

A space–time permutation model was performed in SaTScan (v10.0.2, Kulldorff, M. and Information Management Services, Boston and Calverton, MD, USA) to identify significant space–time clusters of cases. This model is appropriate to use where only case data are available and control or population data are unavailable, as was true for the present study [24]. Generally, this technique involves computing a cylindrical window where the circular base corresponds to the spatial area and the height corresponds to the time period. Within each cylinder, the O/E ratio is computed for the proportion of expected number of cases, assuming a homogenous distribution across space and time, compared to the observed number of cases. Cylinders are moved across the study period, centered on each outbreak location, and the O/E ratio is computed. Temporal and spatial window sizes for the analysis were selected based on visual inspection of O/E ratio plots produced from the Knox test. A temporal window of seven days (also chosen to account for “Monday effects”) and spatial window of ten km were used for the space–time permutation model.

### 2.6. Estimation of Farm-Level Reproduction Ratio (R_0_)

The reproduction ratio (R_0_), or the average number of secondary infections caused by one infected unit throughout the duration of the infectiousness period, was estimated at the country and farm level as the average number of secondary infected farms caused by one infected farm throughout the Dominican Republic [25]. R_0_ > 1 indicates that each infection causes more than one new infection and the outbreak will spread in the population. R_0_ < 1 indicates that each infection causes less than one new infection, and the outbreak will decline. R_0_ = 1 indicates an infectious individual causes only one new case, and the outbreak size does not increase or decrease which can lead to endemicity [26]. The entire Dominican Republic was considered the study area, and an infection was considered as an outbreak farm. R_0_ was estimated using all outbreaks with complete dates (*n* = 153) following the method previously described for use in the absence of population data [27,28]:

(1) $$R_{0}=1+D\times \ln\frac{\frac{C_{t}}{C_{0}}}{t}$$

where D = the duration of the infectious period of the farm (days), C_t_ = the number of cases detected at time t, C_0_ = the number of cases detected at the start of the study period. By considering t as t_d_ = the time to double the number of cases, then C_t_ = 2. Then, Equation (1) is simplified and computed as:

(2) $$R_{0}=1+\frac{D}{t_{d}}\ln2$$

R_0_ was estimated for each doubling interval during the study period. The duration of ASF infection at individual pig level has been estimated to be highly variable depending on virus strain, isolate, and experimental procedures used. Estimates of the minimum and maximum duration of the infectious period (D) for an individual pig range from two to 40 days [29,30,31]. Considering that the infectious period on the farm level may differ greatly from the individual pig level and have high variability due to various factors (size of farm, time of detection and depopulation, etc.), R_0_ was estimated across a range of values of D for an individual farm from one to 40 days. A locally estimated scatterplot smoothing (LOESS) line was fit in R using the package ggplot2 to each series to visualize the trends.

## 3. Results

### 3.1. Descriptive Factors

The dataset contained 155 outbreaks, of which 121 were from farms classified as backyard farms (78%), 30 were from CNT farms (19%), four were ST farms (3%) and none were technical farms. The total number of pigs reported (alive or dead) was 18,910. Despite being the largest proportion of farms, only 4% (857 pigs) of all pig inventory was in backyard farms; conversely, the four ST farms contained 49% (9234) of total pigs (Table 1). The remaining 47% of pigs (8819) were on CNT farms. The median total number of pigs for backyard, CNT, and ST farms was five, 72, and 1974, respectively. Considering all pigs, 97% (18,368) were reported as still alive at the farm visit. By farm type, 74% of backyard pigs, 96% of CNT pigs, and 100% of ST were reported alive.

Clinical signs and/or necropsy findings were reported for 103 unique farms (66%) for one or more production groups present on the farm (Table 2). This information was not recorded for 52 farms in the dataset. Of those farms where clinical presentation was recorded, dead pigs, anorexia/off-feed, and fever were the most reported signs. This order was consistent for piglets and sows, while for nursery, boars, and fattening groups, they were ordered, in highest to lowest frequency, as dead, fever, and anorexia. Diarrhea, lethargy, red skin, and vomiting were also frequently reported across farm types. On eight unique farms, no symptoms were reported in some groups.

The biosecurity measures present varied by farm type (Table 3). No backyard farms reported the presence of measures related to farm access, barn access, staff and personnel protocols, pig breeding, and general farm management, and a minority had closed farm entrances, an unloading ramp for pigs, a perimeter fence in good condition, dedicated carts and work items for each barn, a cement floor with disinfection, good pen drainage, a dedicated feed truck, and used one needle per pig for vaccines and injections. No CNT farms reported having a clean/dirty line to the farm, a visitor log, a vehicle disinfection arch, disinfection of goods and equipment onto the farm, training for staff, or staff having their own pigs. Less than half reported having one or more of 22 different measures related to farm and barn access, staff and personnel protocols, and general farm management. For breeding, about half of backyard farms reported using a live boar, but only 16 reported producing having an exclusive boar for their own use. A higher proportion of CNT farms were reported as using live boars for breeding than backyard farms, but most used exclusive boars. All ST farms reported having an unloading ramp with a clean unloading/loading area, cement barn floors with disinfection, adequate pen drainage, feeders in good condition, using live boars but all with exclusive use or producing their own semen, burying mortality on-farm, and using one needle per pig for injections. The source of water used for pigs was not recorded on any ST farms and most CNT farms, but for backyard farms where it was recorded, the majority used drinking water sources (aqueduct, well, or house water) compared to non-drinking water sources (river or stream).

The reason for the ASF introduction at their farm, proposed by the producers, was summarized into 11 categories (Table 4). A total of 122 farms (79%) provided a suspect reason. The most common reason given, entirely from backyard farms, was close proximity to other known outbreaks and nearby farms. Considering all farm types, movements of pigs between farms or allowing pigs to move free-range was the second most reported reason and first when considering only CNT farms. Swill-feeding, also known as garbage feeding, was the third most cited reason, second overall for backyard farms. Only two of the four ST farms provided a reason, both suggesting movements of herons specifically (grouped under “non-pig animals”). Movements or contact with people, feed (not specifically reported as swill-feeding), sharing breeding boars between multiple farms, and movements or presence of non-pig animals (including dogs, livestock species, birds) were also commonly reported.

### 3.2. Descriptive Spatiotemporal Analysis

**Figure 1:**
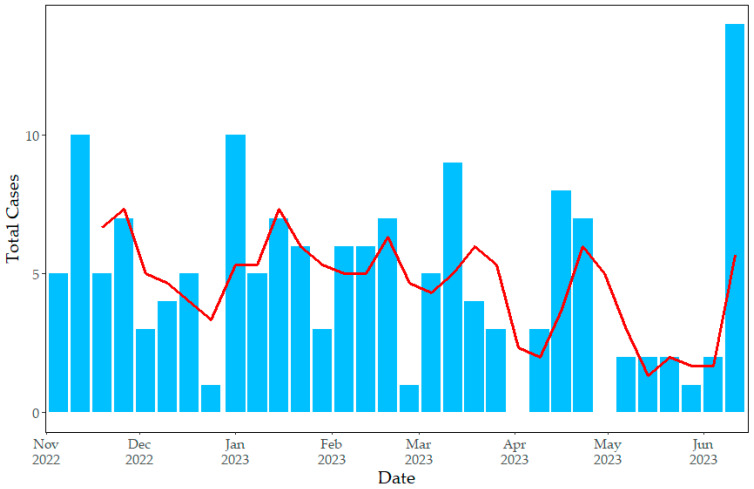
Weekly number of African Swine Fever outbreaks (blue bars) and three-week moving average (red line) reported in the Dominican Republic from 11 November 2022 to 17 June 2023.

The number of reported cases by week (Figure 1) varied from zero to 14 with a mean of 4.8 (SD = 3.2) and median of five. The three-week rolling average varied from one to seven (mean = 4.5, sd = 1.7, median = 5). Two one-week periods (2–8 April and 30 April–6 May) had no cases reported. Overall, ASF cases appeared relatively evenly distributed throughout the study period, with no clear trends.

**Figure 2:**
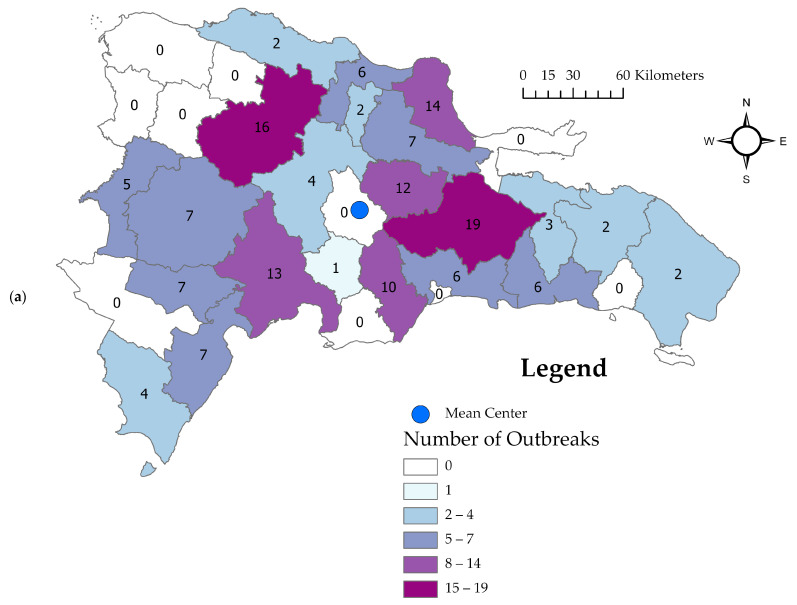

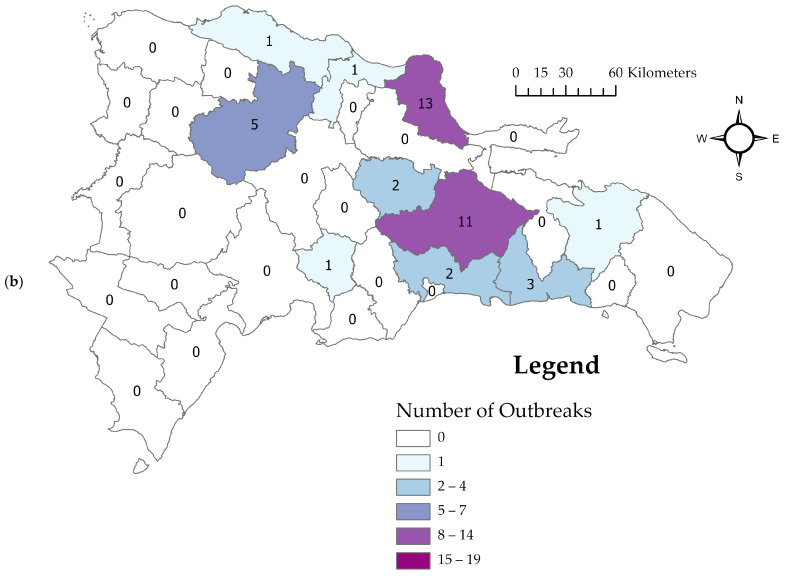

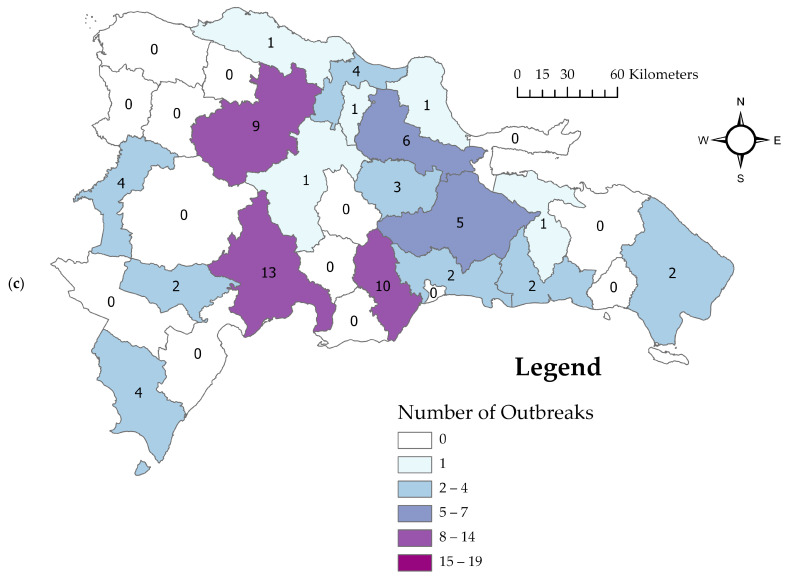

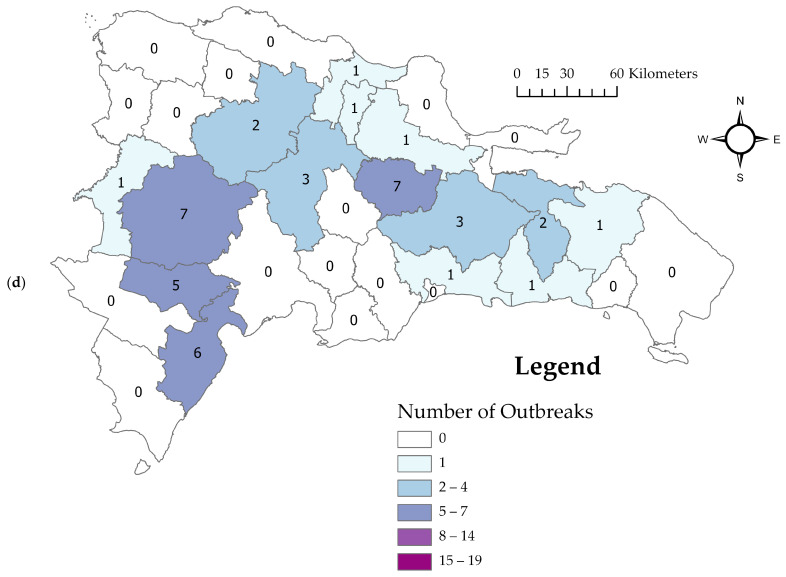
Choropleth maps of (**a**) total number of African Swine Fever outbreaks (*n* = 155 total) in the Dominican Republic reported by province (darker colors represent higher numbers of outbreaks) and overall mean center, and (**b**) number of African Swine Fever outbreaks by province from November to December 2022 (*n* = 40), (**c**) January to March 2023 (*n* = 71), and (**d**) April to June, 2023 (*n* = 42). Note that 1 outbreak from Barahona province and 1 outbreak from Santo Domingo province did not have dates and are only represented in (**a**).

Geographically, cases were mainly located in central regions. The provinces reported a range of 0 to 19 cases. The provinces of Monte Plata (*n* = 19), Santiago (*n* = 16), and María Trinidad Sánchez (*n* = 14) had the highest cumulative number of reported cases across the study period. Ten provinces had no reported ASF cases. The mean center (Figure 2a, blue circle) of all case locations was in the province of Monseñor Nouel.

### 3.3. Between-Farm Reproduction Ratio

**Figure 3:**
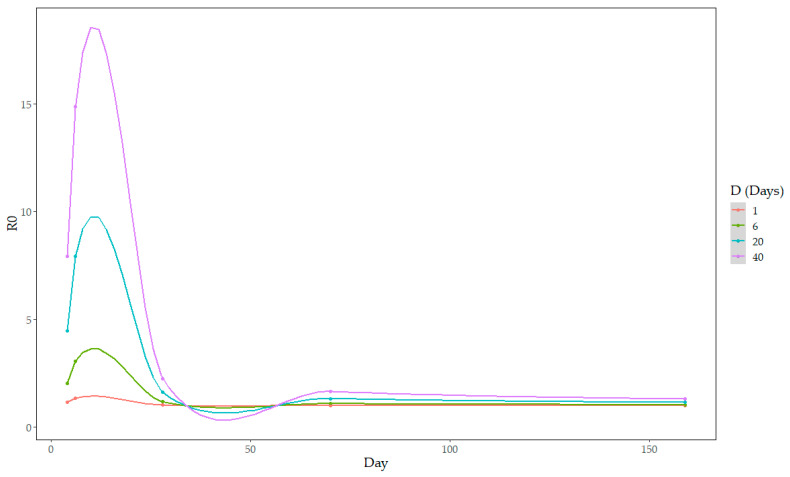
Values of between-herd reproduction ratio (R_0_, points) and their locally estimated scatterplot smoothing estimations (lines) across the study period for different durations of the infectious period of the farm (D).

Five time intervals were identified where the number of infections doubled (Figure 3; at days 4, 6, 28, 70, and 159 of the study period), resulting in five estimations of R_0_. For values of D > 6 days, R_0_ was above two for the first two timepoints (corresponding to the first 6 days of the study period). For values of D < 32 days, R_0_ was estimated at below two by day 28 of the study period. For all values of D, R_0_ was below two for the remaining timepoints and approaching one. For example, in Figure 3, the value of R_0_ at day 159 for D = 1, 6, 20, or 40 days was 1.01, 1.05, 1.16, and 1.31, respectively.

### 3.4. Cluster Analysis

**Figure 4:**
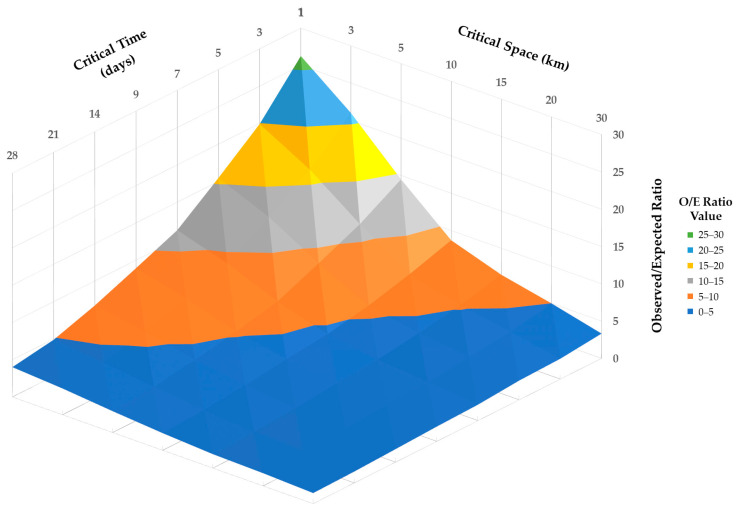
Surface map of observed to expected ratio values (*y*-axis, dark blue = 0—5, orange = 5—10, gray = 10—15, yellow = 15—20, light blue = 20—25, and green = 25—30) from Knox test across combinations of spatial cutoff values (*x*-axis, 1, 3, 5, 10, 15, 20, and 30 km) and temporal cutoff values (*z*-axis, 1, 3, 5, 7, 9, 14, 21, and 28 days).

The Global Moran I’s autocorrelation statistic was significant (I = 0.71, *p* < 0.001), which is consistent with a clustered pattern of cases across the total study period. All combinations of spatial and temporal cutoff values assessed using the Knox test for clustering were significant (*p* < 0.001), supporting clustering of cases across a range of space and time values. O/E ratio values are depicted in Figure 4. O/E ratio values varied from 26.26 (spatial cutoff = 1 km, temporal cutoff = 1 day) to 1.42 (spatial cutoff = 30 km, temporal cutoff = 28 days). A flattening of O/E ratio values was observed with spatial cutoff values of ten km and temporal cutoff values of seven to nine days.

### 3.5. Space–Time Permutation Model

Nine significant space–time clusters (*p* < 0.05) were identified from the space–time permutation model (Table 5). These clusters occurred between January and June 2023 (within 7 day windows). Two clusters were identified in January, two in February, two in March, one in April, and two in June, all in 2023. The number of locations per cluster ranged from four to seven, and the radius of clusters ranged from zero to 2.23 km. Clusters with a radius of zero km involved locations with the same latitude and longitude coordinates as recorded by the veterinary officer at the time of their visit. All locations identified in the clusters were backyard farms.

## 4. Discussion

ASF continues to be an important disease threat globally and to the Dominican Republican swine industry. The present study is one of few currently published sources to evaluate the epidemiological dynamics of ASF spread in the Dominican Republic. These results provide an important description of the ongoing situation that can be used by government officials and industry stakeholders to improve ASF control and management.

The location of cases in relatively central regions of the Dominican Republic (Figure 2) is consistent with pig production in the country and previous reports of ASF outbreaks [14,16,32]. The relatively stable incidence of ASF cases throughout the study period (Figure 1), lacking any clear upward or downward trend, and R_0_ values approaching 1 (Figure 3) are consistent with endemic patterns of disease [26]. The amount of data was insufficient to identify clear temporal trends, such as seasonal analysis, or geographic spread. The R_0_ values here are consistent with between-herd field estimates in domestic pigs from Uganda (ranging from 1.58 to 3.24 depending on the calculation method used) [33], Ukraine (1.65) [34], and the Russian Federation (2–3) [35]. However, it should be noted that the estimation of R_0_ is based on assumptions of homogeneous mixing of the population (same number of contacts for all individuals), which may in reality differ based on varying practices by the farm type, and the estimated duration of infectiousness [36], which may be difficult to estimate at farm level. Additionally, underreporting of cases may lead to an underestimation of R_0_, but this can be difficult to assess and could vary over time depending on economic factors and producer awareness.

Results from the Knox test indicate significant clustering of outbreak even when considering small spatial distances (1, 3, and 5 km) and temporal cutoff values (1, 3, 5, 7, and 9 days). In this dataset, this is because many outbreaks were recorded with the same or very similar date and location, likely reflecting a pattern of disease spread where multiple neighboring backyard farms may become infected with ASF nearly simultaneously or in close succession. This is consistent with findings from the space–time permutation model, in which the radius of significant space–time clusters was 2.23 km or less. In four of the clusters, the geographic location of the involved backyard farms was nearly indistinguishable, as represented by the same latitude and longitude coordinates being recorded for those farms (which was validated during data cleaning through discussions with data collectors). Results from the spatiotemporal and cluster analyses and observations of biosecurity measures and producer behaviors may indicate the need to redefine the epidemiological unit in settings where farms are located adjacent or overlapping one another, and management practices lead to the common sharing of land, animals, feed, and other potential disease introduction sources. Practically, the unit may be better defined as a geographic area, allowing for improved management by veterinary authority and epidemiologists.

The clinical signs and necropsy findings present on some farms are consistent with the previously reported presentation for ASF in the Dominican Republic. In previous works in 2021 and 2022, a heterogenous presentation of ASF was reported, from acute, hemorrhage disease in some outbreaks to a more subacute and nonspecific disease on other farms [32,37]. Of the 103 farms that had clinical sign information in this dataset, mortality, anorexia, and fever were the most reported signs across production types. Eight farms had asymptomatic infections reported. All reported hemorrhagic signs combined (red skin, epistaxis, unspecified hemorrhage) were the fourth most reported symptom. Almost no necropsy signs were reported, but this is likely because conducting a necropsy was not part of the official control program or case definition used to verify these outbreaks.

Despite reported mortalities, a relatively large number of pigs were reported as still alive on farms at the time of depopulation for indemnity purposes (Table 1). This could be due to early reporting of ASF outbreaks, which may be encouraged if incentivized through indemnity and other support. Another possibility is that the reported heterogenous presentation may result in outbreaks with lower mortality, but this is still unknown. Surviving but infected pigs that are not reported may be more likely to be moved or sent to slaughter, especially in cases where producers may perceive a slow or insufficient indemnity response by government officials or not wish their farm to be depopulated, which ultimately further drives the spread of ASF. Understanding the factors behind this high number of live pigs may help to reduce the risk of infected pigs being dispersed.

The biosecurity measures present varied considerably across farm types. As expected, backyard farms had fewer biosecurity measures present, such as lacking secure perimeters for farms and barns, protocols and training for farm workers (which in the case of backyard farms, are likely the owners themselves), consistent cleaning and disinfection, and protocols for breeding and animal management. CNT farms had more measures present compared to backyard farms, such as an increased percentage of farms reporting having showers, truck disinfection, animal loading and unloading areas with ramps, separation via clean-dirty lines and closed farm entrances, dedicated equipment, and pest control. ST farms had the most biosecurity measures present. This may contribute to the low number of reported outbreaks in ST farms, but this is difficult to assess without population data for each farm type. Overall, lack of biosecurity measures likely contributes significantly to the continued spread of ASF, especially through closely and densely located backyard farms.

The reason for their ASF introduction, as suspected by producers, is mostly consistent with known risks for ASF spread [1,6]. It should be noted that these responses do not reflect the results of outbreak investigations conducted by veterinary officials, but they do provide an indication of producers’ behaviors and management actions. Being near a known outbreak or other farms was the most commonly listed reason (Table 4, 35% of 122 respondents) and solely by backyard farms. This is consistent with results from cluster analysis and space–time permutation model, where small spatial distances resulted in significant clusters with small radiuses. Other management practices, such as moving pigs, swill-feeding, and sharing breeding boars, were also frequently mentioned by producers, and providing key messaging and support targeting these behaviors will help to support improved biosecurity and management, ultimately to limit ASF spread.

Under-reporting may be an important limitation for this data set, but this is difficult to estimate or quantify. Case reporting amongst some producers in the Dominican Republic may be largely driven by economic factors to reduce financial losses from ASF outbreaks. Producers may compare the sale price amongst carnicerias, more informal meat markets and butcher shops as compared to slaughter plants, to indemnity rates provided after government-sponsored depopulation. Higher prices from carnicerias may incentivize producers to sell infected pigs before high mortality from ASF occurs, while when sale prices decrease, higher indemnity rates may be preferred. A similar effect of indemnity rates and market price on passive surveillance has been reported in Vietnam [38]. Similarly presenting swine diseases may also play a role in underreporting. Because other diseases such as Classical Swine Fever and Porcine Reproductive and Respiratory Syndrome (PRRS) are also present in the Dominican Republic, small backyard farms may simply dispose of dead pigs and repopulate without suspecting ASF. Other factors that have been previously described to affect passive reporting of veterinary diseases by farmers include producer knowledge and awareness of the disease and reporting systems, uncertainty about when to report, mistrust of veterinary authorities, and fear of social consequences of reporting [39]. The current analysis was also limited by the lack of control or population data, which is hard to access or not readily available in the Dominican Republic swine industry, so a risk factor analysis could not be performed. Future work should aim to collect uninfected population data for further analysis and comparison. Finally, not all farms had all information reported, such as clinical signs or suspected introduction reasons, resulting in a smaller sample size for those variables.

In summary, these results provide a recent update on spatiotemporal dynamics of ASF in the Dominican Republic and ongoing practices of pig holders that drive ASF spread. The information reported here will help swine industry stakeholders and government officials in developing ASF control strategies. Outbreaks were primarily centrally located within the country and backyard farms, though the vast majority of individual affected animals were located in the 30 CNT and 4 ST farms. Clustering was high due to many neighboring backyard farms being detected with ASF almost simultaneously. Future works with access to control or population data will allow for more investigation into the specific, important risk factors present in the Dominican Republic and provide further useful information for developing prevention and control strategies.

## Figures and Tables

**Table 1:** Inventory of all, live, and dead pigs on backyard (*n* = 121), commercial non-technical (CNT, *n* = 30), and ST (semi-technical, *n* = 4) farms. SD = standard deviation of the mean.

Inventory Type	Farm Type	Total Number (% of All Pigs)	Mean	SD	Median	Minimum	Maximum
All Pigs	Backyard	857 (4)	7	6.2	5	1	24
	CNT	8819 (47)	294	564	72	28	2855
	ST	9234 (49)	2309	1439	1974	1015	4271
Live Pigs	Backyard	631 (3)	5	5.1	3	0	24
	CNT	8503 (45)	283	552	72	12	2811
	ST	9234 (49)	2309	1439	1974	1015	4271
Dead Pigs	Backyard	226 (1)	2	3.7	0	0	18
	CNT	316 (1)	11	22	0	0	96
	ST	0 (0)	0	0	0	0	0

**Table 2:** Clinical signs and necropsy findings reported by farms (*n* = 103 unique farms) by production stage. “No symptoms” refers to farms where “asymptomatic” or “no signs” was reported and does not represent missing information.

Clinical Sign or Necropsy Finding	Production Type (Number of Farms) * Number Reporting Sign (% By Production Stage) **					
	Piglet (63)	Nursery (35)	Boars (39)	Sows (60)	Fattening (40)	Total
Dead	26 (41)	22 (63)	19 (49)	26 (43)	11 (28)	104 (44)
Anorexia	25 (40)	11 (31)	10 (26)	24 (40)	20 (50)	90 (38)
Fever	12 (19)	14 (40)	11 (28)	20 (33)	16 (40)	70 (30)
Diarrhea	12 (19)	6 (17)	7 (18)	11 (18)	7 (18)	43 (18)
Lethargy	9 (14)	7 (20)	4 (10)	13 (22)	10 (25)	39 (17)
Red skin	5 (8)	5 (14)	5 (13)	4 (7)	4 (10)	30 (13)
Epistaxis	4 (6)	3 (9)	5 (13)	8 (13)	3 (8)	23 (10)
No symptoms	3 (5)	0 (0)	1 (3)	4 (7)	0 (0)	8 (3)
Sudden death (specifically)	4 (6)	0 (0)	2 (5)	1 (2)	0 (0)	7 (3)
Vomiting	1 (2)	0 (0)	2 (5)	3 (5)	1 (3)	7 (3)
Hemorrhage (unspecified)	1 (2)	1 (3)	0 (0)	1 (2)	0 (0)	5 (2)
Nervous signs	1 (2)	0 (0)	0 (0)	1 (2)	2 (5)	4 (2)
Splenomegaly	0 (0)	0 (0)	0 (0)	2 (3)	1 (3)	3 (1)
Respiratory	0 (0)	0 (0)	0 (0)	0 (0)	1 (3)	1 (0.4)

* Number of farms sums greater than 103 due to some farms reporting multiple production types. ** Percentage by farm type of findings reported as percentage of production type reporting the presence of that finding. This does not sum to 1 due to most farms reporting multiple findings.

**Table 3:** Biosecurity measures present and absent by farm type, CNT = commercial non-technical, ST = semi-technical.

Category	Biosecurity Factor	Number (%)		
		Backyard	CNT	ST
Farm Access	Clean/dirty line with change of clothes and boot covers	0 (0)	0 (0)	2 (50)
Farm Access	Has a shower	0 (0)	5 (17)	3 (75)
Farm Access	Has closed entrance doors	2 (2)	10 (33)	3 (75)
Farm Access	Has visitor log	0 (0)	0 (0)	1 (25)
Farm Access	Has vehicle disinfection arch	0 (0)	0 (0)	1 (25)
Farm Access	Has foot baths and change of disinfectant	0 (0)	1 (3)	1 (25)
Farm Access	Disinfects goods and equipment brought on farm	0 (0)	0 (0)	3 (75)
Farm Access	Has unloading ramp	1 (0.8)	10 (33)	4 (100)
Farm Access	Perimeter fence is in good condition	1 (0.8)	4 (13)	3 (75)
Barn Access	Has cleaning and disinfection of the entrance	0 (0)	4 (13)	3 (75)
Barn Access	Each barn has dedicated staff	0 (0) *	6 (20)	3 (75)
Barn Access	Each barn has dedicated carts and work items	1 (0.83)	4 (13)	3 (75)
Barn Access	Has rodent control	0 (0)	2 (7)	3 (75)
Barn Access	Has bird curtain	0 (0)	5 (17)	1 (25)
Barn Access	Has clean staff clothing	0 (0)	1 (3)	2 (50)
Barn Access	Has footbath with frequent change of disinfectant	0 (0)	1 (3)	1 (25)
Barn Access	Has cement floor with periodic disinfection	6 (5.0)	12 (40)	4 (100)
Barn Access	Has feeders in good condition and periodic disinfection	0 (0)	6 (20)	4 (100)
Staff	Staff receive biosecurity training	0 (0) *	0 (0)	2 (50)
Staff	Staff have their own pigs	0 (0) *	0 (0)	1 (25)
Staff	Staff have dedicated and clean clothes	0 (0) *	2 (7)	3 (75)
Staff	Staff required to shower upon entering the barn or pig house	0 (0) **	3 (10)	3 (75)
Staff	Staff bring food into the barns	32 (27) *	26 (87)	3 (75)
Breeding	Use natural mount	67 (55)	26 (87)	4 (100)
Breeding	Use artificial insemination	0 (0)	1 (3)	3 (75)
Breeding	Produces their own semen	25 (21)	21 (70)	4 (100)
Breeding	Has boars for exclusive use	16 (13)	22 (73)	4 (100)
Breeding	Produces their own replacement pigs	28 (23)	21 (70)	3 (75)
General	Buries mortality on farm	34 (28)	21 (70)	4 (100)
General	Feed enters with its own truck	9 (7)	16 (53)	3 (75)
General	Trucks are disinfected at the entrance and exit	0 (0)	4 (13)	3 (75)
General	Other species are present on premise	27 (23) *	18 (60)	1 (25)
General	Uses one needle per pig for injections of drugs or vaccines	7 (6)	5 (17)	4 (100)
General	Has weeds on the perimeter or close to the food	62 (51)	18 (60)	0 (0)
General	Pens drain adequately without causing flooding	1 (0.8)	5 (17)	4 (100)
General	Has clean animal loading and unloading area	0 (0)	6 (20)	4 (100)
Water source for pigs ***	Drinking water	67 (55)	10 (33)	***
	Non-drinking water	4 (3)	0 (0)	***

* 1 missing response. ** 2 missing responses. *** Missing responses were 44 (backyard), 26 (CNT), and 4 (ST).

**Table 4:** Summary of reasons proposed by producers for introduction of African Swine Fever (ASF) at their farm (*n* = 122), by farm type (CNT = commercial non-technical, ST = semi-technical).

Reason for ASF Introduction Suspected by Producer	Number of Farms (%) *			
	Backyard (*n* = 103)	CNT (*n* = 17)	ST (*n* = 2)	Total (*n* = 122)
Proximity to other outbreaks and farms	43 (42)	0 (0)	0 (0)	43 (35)
Movements of pigs between farms or free-range domestic pigs	26 (25)	8 (47)	0 (0)	34 (28)
Swill or garbage feeding	32 (31)	0 (0)	0 (0)	32 (26)
Movements of and contact with people	10 (10)	4 (24)	0 (0)	14 (12)
Feed (unspecified source)	11 (11)	2 (12)	0 (0)	13 (11)
Sharing breeding boar	8 (8)	4 (24)	0 (0)	12 (10)
Non-pig animals	5 (5)	3 (18)	2 (100)	10 (8)
Water	2 (2)	1 (6)	0 (0)	3 (3)
Vehicles/Trucks	0 (0)	2 (12)	0 (0)	2 (2)
Carcass disposal method	1 (1)	0 (0)	0 (0)	1 (0.8)
Near landfill	1 (1)	0 (0)	0 (0)	1 (0.8)

* Percentage calculated as the number of farms reporting that reason out of that farm type or all (*n* = 122) that reported a suspected reason. Percentages do not sum to one, due to some farms reporting multiple suspected reasons.

**Table 5:** Significant clusters (*p* < 0.05) of African Swine Fever outbreaks reported in the Dominican Republic between 11 November 2022 and 17 June 2023.

Cluster	Radius (km)	Timeframe	Number of Cases	O/E Ratio	*p*-Value
1	0.76	1 January 2023 to 7 January 2023	6	15.3	<0.001
2	2.23	22 January 2023 to 28 January 2023	6	21.3	<0.001
3	0.00042	11 June 2023 to 17 June 2023	7	10.9	<0.001
4	0.21	5 March 2023 to 11 March 2023	4	30.6	<0.001
5	0	5 February 2023 to 11 February 2023	4	25.5	0.001
6	0	12 February 2023 to 18 February 2023	4	25.5	0.001
7	0.63	23 April 2023 to 29 April 2023	4	21.9	0.003
8	0	12 March 2023 to 18 March 2023	4	17	0.011
9	0	11 June 2023 to 17 June 2023	5	10.9	0.014

## Data Availability

Data are the property of the Dominican Republic government and have been shared with us to conduct the research here to support ASF control activities in the country. Any requests should be directed to the Dominican Republic Incident Command for ASF.

## Supplementary Materials

The following supporting information can be downloaded at: https://www.mdpi.com/article/10.3390/pathogens12121414/s1, File S1: Questionnaire used by the Dominican Republic Ministry of Agriculture veterinary officers for data collection and verification of passively reported African Swine Fever farm outbreaks.
